# *ORMDL3*: from an asthma susceptibility gene to multi-disease associations

**DOI:** 10.3389/fgene.2026.1720829

**Published:** 2026-03-18

**Authors:** Zichao Han, Siyi Guo, Chao Wang, Yewen Niu, Jiayi Liu, Kaifeng Li, Dong Li, Fei Yu, Xuan Li

**Affiliations:** 1 The People’s Hospital of ZouPing City, Binzhou, Shandong, China; 2 Zibo Hospital of Traditional Chinese Medicine, Zibo, Shandong, China; 3 Zibo Central Hospital, Zibo, Shandong, China; 4 Shandong University of Traditional Chinese Medicine, Jinan, Shandong, China

**Keywords:** asthma, gene, multi-disease associations, ORMDL3, review

## Abstract

The ORMDL3 protein, encoded by the *ORMDL3* gene, functions as a transmembrane protein in the endoplasmic reticulum. Initially identified through its genetic link to asthma susceptibility, ORMDL3 plays a key role in regulating sphingolipid metabolism by inhibiting Serine Palmitoyltransferase activity. This regulation influences the synthesis of bioactive lipids like ceramides, which in turn affect cellular homeostasis, inflammatory responses, and immune regulation. Recent studies show that ORMDL3’s function extends beyond the respiratory system, with involvement in obesity, diabetes, atherosclerosis, inflammatory bowel disease, autoimmune diseases, and various cancers. It regulates processes such as endoplasmic reticulum stress, the unfolded protein response, autophagy, calcium signaling, and inflammatory pathways. This review highlights ORMDL3’s expression patterns, molecular mechanisms, and clinical relevance across different diseases, emphasizing its role as a biological node linking multiple pathologies. Additionally, it discusses ORMDL3’s potential as a target for innovative therapies and diagnostic biomarkers, laying the groundwork for future interdisciplinary research and precision medicine applications.

## 1 Introduction

ORMDL3 is a transmembrane protein in the ER, encoded by a gene located in the human chromosome 17q21 region (Nowakowska et al., 2023). As a key member of the ORMDL protein family, which also includes ORMDL1 and ORMDL2, ORMDL3 shares substantial sequence homology with its counterparts but likely performs distinct functional roles within cellular processes (Demkova et al., 2023). Early studies revealed that ORMDL3 primarily regulates sphingolipid metabolism, acting as a negative regulator of SPT. By modulating SPT activity, ORMDL3 helps maintain sphingolipid homeostasis, which is essential for processes such as signal transduction, membrane integrity, and intercellular communication. Thus, ORMDL3’s regulation of sphingolipid metabolism plays a significant physiological role (Chen et al., 2024). The protein gained prominence following the identification of the *ORMDL3* gene as a susceptibility locus for asthma. A pivotal 2007 GWAS by Moffatt et al. first demonstrated a strong correlation between SNPs in the *ORMDL3* gene and an increased risk of childhood asthma (Moffatt et al., 2007). Subsequent studies across diverse ethnic populations consistently confirmed this association, solidifying the link between *ORMDL3* genetic variation and asthma susceptibility (Gautam et al., 2020). ORMDL3 expression is significantly upregulated in asthma, where it contributes to disease pathogenesis by activating inflammatory pathways, promoting excessive mucus production, and inducing structural airway remodeling (Cheng et al., 2019). In recent years, researchers have expanded the focus to ORMDL3’s role in diseases beyond asthma. Studies suggest its involvement in IBD, metabolic disorders, autoimmune conditions, and cancer (Malicevic et al., 2024; Yang et al., 2019; Verlaan et al., 2009; Zeng et al., 2025). The connection between ORMDL3 and multiple diseases points to shared mechanisms, such as regulating the UPR in the ER, disrupting sphingolipid homeostasis, and influencing calcium signaling. These key pathways position ORMDL3 as a central hub involved in various diseases. These pathways position ORMDL3 as a central hub in various diseases. However, much of the research is still in its early stages, and the precise functions, mechanisms, and clinical relevance of ORMDL3 across these diseases remain unclear. This review aims to provide a comprehensive summary of the current research on ORMDL3 in these diseases, explore its potential clinical applications, and suggest promising directions for future research.

## 2 Classical functions of ORMDL3

This section examines the functions of ORMDL3, providing a detailed analysis of its key roles in asthma and cellular physiological processes. ORMDL3, a membrane protein in the ER, primarily regulates lipid metabolism, with a particular focus on sphingolipid synthesis. By modulating lipid balance within the ER, ORMDL3 influences cellular signaling and membrane stability. Additionally, it plays a crucial role in ERS. Under stress conditions, ORMDL3 regulates protein folding and degradation, helping to maintain cellular homeostasis. Recent research has revealed that ORMDL3 also plays a role in immune responses and the development of inflammatory processes. These findings provide a solid theoretical foundation for later chapters exploring ORMDL3’s involvement in diseases affecting other systems.

### 2.1 Regulation of sphingolipid metabolism

**FIGURE 1:**
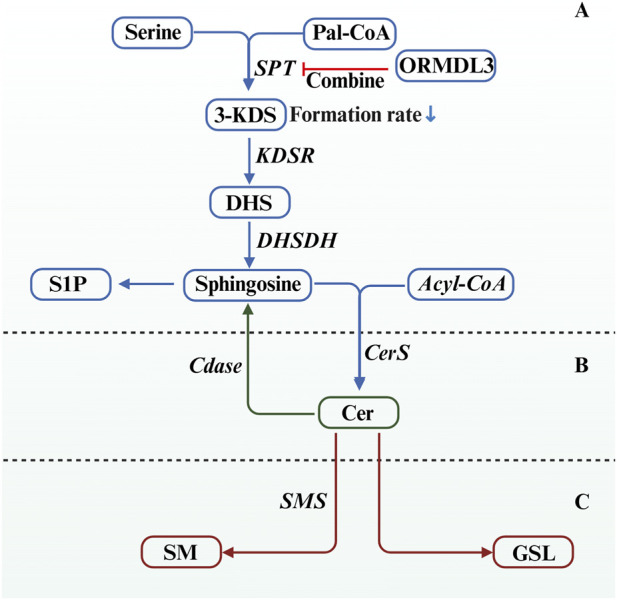
Sphingolipid synthesis and the Intervention of ORMDL3. **(A)** This stage represents the *de novo* synthesis of sphingolipids, beginning with the formation of 3-KDS from serine and Pal-CoA catalyzed by SPT, followed by a series of reactions to generate Sphingosine. **(B)** This stage involves the formation of Cer (the central metabolic intermediate in sphingolipid biosynthesis) from sphingosine and acyl-CoA catalyzed by CerS. **(C)** At this stage, Cer can serve as a precursor for the continued synthesis of SM and GSL. * ORMDL3 combines with SPT, thereby reducing the production rate of 3-KDS and inhibiting the *de novo* synthesis of sphingolipids.

Sphingolipid metabolism is crucial for maintaining cellular membrane integrity and signal transduction. The process begins with the conjugation of Serine and Pal-CoA, catalyzed by SPT to form 3-KDS, which is then reduced by a KDSR to produce DHS. This compound is converted into sphingosine by DHSDH and, together with Acyl-CoA, undergoes acylation catalyzed by CerS, introducing a fatty acyl chain to form Cer—the central metabolic intermediate in sphingolipid biosynthesis (Schorsch et al., 2012). Cer plays a pivotal role in sphingolipid metabolism and can be further processed into bioactive sphingolipids. SM is predominantly found in the outer leaflet of the plasma membrane, contributing to membrane structure, while GSL are involved in cellular recognition and signal transduction. Additionally, Cer can be deacylated to generate sphingosine, which is subsequently phosphorylated to form S1P. S1P acts as a critical signaling lipid that regulates immune cell migration, angiogenesis, and inflammatory responses (Hannun and Obeid, 2018). Located on the ER membrane, ORMDL3 functions as a key regulatory protein that binds to and inhibits the SPT complex, thereby reducing the production rate of 3-KDS (For details, see Figure 1). This mechanism represents an essential feedback inhibition pathway in sphingolipid biosynthesis, particularly during the initial stages (Gupta et al., 2015). As cellular sphingolipid levels increase, particularly Cer accumulation, ORMDL3 activity or stability is enhanced, which strengthens the inhibition of SPT and reduces the synthesis of new sphingolipid precursors to prevent excessive accumulation. In contrast, when sphingolipid levels are low, ORMDL3-mediated inhibition decreases, leading to increased SPT activity and greater sphingolipid production to restore cellular homeostasis. In asthma, ORMDL3 overexpression inhibits SPT, resulting in lower sphingolipid levels, which disrupt cell membrane integrity, induce ERS, and promote the release of Th2-type inflammatory cytokines. These changes contribute to airway hyperresponsiveness, excessive mucus production, and bronchial remodeling (Luthers et al., 2020). Additionally, ORMDL3 may influence immune cell migration through its effect on S1P signaling, further explaining its pathogenic role in asthma and highlighting a potential target for therapeutic strategies (Kim et al., 2020).

### 2.2 ERS, the UPR, and calcium signaling

The ER is a crucial organelle responsible for protein synthesis, folding, maturation, and calcium storage. Overexpression of ORMDL3 inhibits SPT activity, reducing sphingolipid levels and disrupting lipid homeostasis, which can induce ERS. This stress response activates the UPR and disrupts calcium signaling, creating an integrated regulatory network (Celik et al., 2023). ERS can be triggered by various internal and external factors, including hypoxia, nutrient deficiency, impaired protein folding, and disrupted calcium homeostasis. It is mainly characterized by the accumulation of unfolded or misfolded proteins within the ER lumen, along with disturbances in calcium balance and redox equilibrium (Groenendyk et al., 2021). As an adaptive response to ERS, the UPR functions through three major signaling pathways, mediated by the transmembrane sensors PERK, IRE1α, and ATF6. Upon activation, PERK phosphorylates eIF2α, which attenuates global protein synthesis to reduce the burden on the ER. IRE1α splices XBP1 mRNA, leading to the upregulation of Molecular chaperone and ERAD components, thereby enhancing protein folding and clearance. ATF6 is cleaved in the Golgi apparatus, activating the transcription of Molecular chaperone, ERAD factors, and XBP1, which directly boosts folding capacity and provides additional substrates for IRE1. Together, these pathways collaborate to restore ER homeostasis (Chen et al., 2023). However, prolonged or excessive UPR signaling can promote inflammation. For instance, IRE1α activates JNK and p38 MAPK pathways, which promote M1 macrophage polarization (Guo et al., 2025). Overexpression of ATF4 and CHOP can worsen inflammatory responses and trigger apoptosis (Ji et al., 2025). Ultimately, UPR pathways activate key pro-inflammatory transcription factors like NF-κB and AP-1, leading to the production and release of cytokines such as TNF-α, IL-1β, IL-6, and IL-8 (Rufo et al., 2022). In asthma pathogenesis, ORMDL3 overexpression inhibits sphingolipid synthesis, induces ERS, and activates the UPR, driving it toward pro-inflammatory and pro-apoptotic responses. This cascade promotes the release of Th2 cytokines and plays a key role in the disease’s pathological processes (Han et al., 2025). The ER also serves as the primary calcium reservoir within the cell, and its calcium homeostasis is essential for normal cellular function. ERS is often linked to disrupted calcium signaling, where the function of calcium release channels, such as the IP3R and RyR, is impaired, causing abnormal calcium efflux from the ER into the cytoplasm (Vecchi et al., 2024). Simultaneously, the SERCA activity decreases, limiting calcium reuptake into the ER. These changes lead to increased cytosolic calcium concentrations and depletion of ER calcium stores (Toprak, 2026). Dysregulated calcium signaling affects UPR activity through multiple mechanisms, such as impaired Molecular chaperone function, which hampers protein folding and processing (Kopp et al., 2019). Calcium dysregulation also alters the phosphorylation levels of PERK and IRE1, interfering with their downstream signaling (McCarthy et al., 2020). Additionally, persistently high cytosolic calcium levels can activate calpains, which cleave various protein substrates and disrupt cellular function. Elevated calcium also causes mitochondrial calcium overload, impairing energy metabolism and generating excessive Reactive Oxygen Species, which accelerates apoptotic cell death (Cui et al., 2022).

### 2.3 Autophagy

**FIGURE 2:**
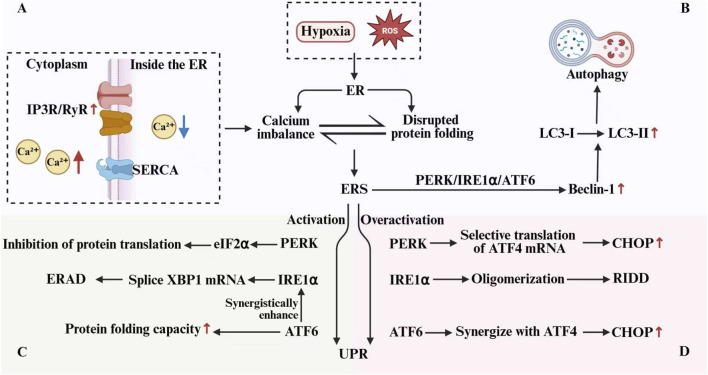
ERS, UPR, Autophagy, and Calcium Signaling. **(A)** Impaired IP3R/RyR and SERCA function causes ER calcium store depletion, which, together with disrupted protein folding, triggers ERS. **(B)** ERS triggers autophagy activation, increasing the expression of the autophagy marker Beclin-1 and promoting the conversion of LC3-I to LC3-II. As an adaptive response to ERS, the UPR functions through PERK, IRE1α, and ATF6. Moderate UPR activation exerts a protective effect **(C)**, whereas excessive activation drives it toward pro-apoptotic outcomes **(D)**.

Autophagy is a cellular process where damaged organelles, protein aggregates, or other unnecessary components are engulfed by a double-membraned autophagosome. This vesicle then fuses with a lysosome, and its contents are degraded by lysosomal hydrolases, allowing for the recycling of cellular components (Li J. et al., 2025). IIn asthma, both ERS and autophagy are hyperactivated. ERS triggers autophagy activation, increasing the expression of the autophagy marker Beclin-1 and promoting the conversion of LC3-I to LC3-II. These changes may contribute to airway epithelial damage and enhanced airway responsiveness (The above mechanisms are shown in Figure 2) (Qian et al., 2025; Dastghaib et al., 2021). ORMDL3 overexpression induces a significant increase in autophagosome formation and upregulates autophagy-related proteins such as LC3B, ATG3, ATG7, and ATG16L1. ORMDL3 interacts with SERCA2, impairing intracellular calcium mobilisation and inhibiting calcium influx. This disruption in calcium homeostasis promotes autophagy and may trigger cell death. In bronchial epithelial cells derived from asthma patients, ORMDL3 expression levels correlate significantly with multiple autophagy-related genes. These findings suggest that ORMDL3 activates autophagy by disturbing calcium homeostasis, potentially leading to functional impairment of bronchial epithelial cells and contributing to asthma pathogenesis (Guo et al., 2022). Furthermore, Li et al. demonstrated that ORMDL3 overexpression upregulates key ERS-UPR factors (e.g., SERCA2b and ATF6) and autophagy-related proteins (e.g., Beclin 1 and LC3BII). Knocking down ATF6 or inhibiting autophagy reversed the impaired degranulation and cytokine/chemokine production caused by ORMDL3. These results suggest that ORMDL3 suppresses antigen-mediated mast cell activation via ATF6 UPR signaling and autophagy, reducing allergic responses and highlighting a potential target for mast cell-related diseases (Li et al., 2021).

### 2.4 Regulation of airway inflammation and remodeling

Airway remodeling refers to structural changes in the airway wall that go beyond normal physiological repair. These alterations occur in chronic inflammatory airway diseases due to persistent inflammation and repeated tissue injury and repair. Key features include excessive ECM deposition, airway smooth muscle proliferation and hypertrophy, angiogenesis, and epithelial barrier dysfunction. Together, these processes result in airway wall thickening and progressive functional decline. They form the core pathological basis for disease progression, refractory symptoms, and worsening lung function (Jakiela et al., 2025). ORMDL3 contributes to the regulation of inflammatory responses through several mechanisms. For example, it significantly increases the expression of IL-4, TNF-α, and MCP-1 after FcεRI cross-linking. An S1P agonist can inhibit this effect, suggesting that ORMDL3 may regulate pro-inflammatory cytokine expression via the S1P pathway (Ogi et al., 2021). In human airway epithelial cells (A549 and NHBE), ORMDL3 overexpression under poly I:C stimulation promotes the release of IL-6 and IL-8 (Laura et al., 2021). On the other hand, silencing ORMDL3 reduces IL-6 and IL-8 release independently of steroids and alleviates IL-1β-induced ERS (Zhang et al., 2019). Additionally, ORMDL3 suppresses JNK1/2–MMP-9 pathway activation, significantly reducing airway hyperresponsiveness, airway inflammation, mucus hypersecretion, and peribronchial collagen deposition (Wang et al., 2019). Furthermore, ORMDL3 activates the ERK/MMP-9 pathway, which markedly increases the levels of various inflammatory cytokines, including TNF-α, IL-4, IL-5, and IL-6, driving asthma-related inflammation (Duan et al., 2020; Wu et al., 2018). Notably, the expression of p-ERK and MMP-9 strongly correlates with bronchial wall thickness. Since ORMDL3 expression is also positively correlated with p-ERK and MMP-9, it may promote pathological airway remodeling in asthma patients by activating the p-ERK/MMP-9 pathway (Yu et al., 2017). Additionally, ORMDL3 expression is linked to the progression of EMT *in vivo* (Cheng and Shang, 2018). Through these coordinated pathways, ORMDL3 perpetuates the inflammation-remodeling cycle and accelerates irreversible changes in airway structure.

## 3 ORMDL3 in non-asthma

This section explores the multifaceted roles of ORMDL3 in human diseases from a multi-system perspective. ORMDL3 plays a key pathophysiological role through a central network. It disrupts ER calcium homeostasis, inducing ERS, which acts as a primary driver. ERS activates downstream signaling pathways, such as the UPR, significantly enhancing the activity of pathways like p38 MAPK and promoting chronic inflammatory responses. Simultaneously, the disruption of calcium signaling works with ERS to precisely regulate the autophagy process. These pathways, including ERS, calcium signaling, autophagy, and inflammation, are not isolated but form an interconnected and mutually regulatory network. While ORMDL3 has traditionally been studied in the context of childhood asthma and repirsatory diseases, where it is recognized as a key regulator of airway inflammation, hyperresponsiveness, and ERS, emerging evidence suggests that its pathophysiological functions extend well beyond the respiratory system. This chapter systematically summarizes recent advancements in understanding ORMDL3’s contributions to endocrine, circulatory, and immune system diseases, as well as cancer. It highlights how ORMDL3 modulates core biological processes such as sphingolipid metabolism, ER homeostasis, and inflammatory signaling in various disease contexts. By integrating this interdisciplinary perspective, we aim to provide a more comprehensive understanding of ORMDL3 as a central molecular node in disease networks and to support its potential as a therapeutic target.

### 3.1 Endocrine system diseases

ORMDL3 acts as a central regulator of sphingolipid metabolism and plays a significant role in the development of metabolic diseases such as obesity and diabetes. GWAS studies confirm that genetic variants of *ORMDL3* are strongly associated with obesity risk. Its expression levels negatively correlate with body mass index, suggesting a role in modulating lipid metabolism and energy balance, thus influencing metabolic health (Pan et al., 2018). Song et al. reported that *Ormdl3* expression is highest in mouse interscapular BAT and significantly lower in specific WAT depots, indicating a potential distinct role for *Ormdl3* in BAT function. Under high-fat diet conditions, *Ormdl3*-deficient mice gain weight faster and develop more severe insulin resistance than wild-type mice. Their adipose tissue shows a marked increase in Cer levels and a suppressed expression of UCP1, a key thermogenic marker. These findings suggest that ORMDL3 regulates sphingolipid synthesis by modulating SPT activity, impairing BAT thermogenesis and WAT browning, and contributing to obesity and metabolic dysregulation (Song et al., 2022). Lee compared *ORMDL* expression in pancreatic islets from lean and obese donors and found a significant reduction in all *ORMDL* mRNAs in obese individuals, with *ORMDL3* showing an approximately 11-fold decrease. In contrast, islets from leptin-deficient obese (ob/ob) mice exhibited significantly higher *Ormdl3* expression than those from lean mice. This may reflect a compensatory mechanism to inhibit SPT activity and reduce ceramide-induced toxicity. Leptin intervention reversed the upregulation of *Ormdl3*, suggesting that leptin helps restore sphingolipid homeostasis through central or peripheral pathways (Lee et al., 2020). Further mechanistic studies show that under high-fat diet conditions, tissues such as adipocytes and the liver accumulate excessive saturated fatty acids, leading to increased Cer synthesis (Klag et al., 2025). Excess Cer acts as a core mediator of lipotoxicity, inhibiting the insulin signaling pathway, causing insulin resistance, and inducing adipocyte apoptosis and inflammation (Razi et al., 2025). Under these conditions, ORMDL3 overexpression reduces Cer production, directly alleviating lipotoxicity and insulin resistance.

Obesity is a major risk factor for various metabolic diseases and serves as a critical pathological basis for the development and progression of diabetes (Brown and Spiegel, 2023). The onset of diabetes is closely linked to the functional state of pancreatic β-cells. β-cell dysfunction can directly lead to insufficient insulin secretion or abnormal secretory patterns, disrupting glucose homeostasis and contributing to the pathophysiology of diabetes (Wang K. et al., 2025). Hurley et al. conducted fasting-refeeding experiments in 12-week-old mice and found that β-cell-specific *Ormdl3* knockout mice showed no changes in C-peptide levels under basal freely-fed conditions, after 6 h of fasting, or upon refeeding. Proinsulin levels during refeeding remained unchanged, and the proinsulin-to-C-peptide ratio was unaffected. Further glucose and insulin tolerance tests revealed no significant abnormalities in glucose tolerance or insulin sensitivity. However, lipidomic analysis showed that under high-fat diet conditions, islets from *Ormdl3*-deficient mice exhibited a significant increase in very long-chain ceramides. In summary, loss of *Ormdl3* specifically in β-cells does not affect systemic metabolism or β-cell function under normal conditions. However, under obesity, *Ormdl3* deficiency leads to a marked increase in specific very long-chain Cer species (Hurley et al., 2023). Holm et al. found that pancreatic islets from patients with T1D had a 77% reduction in sulfatide content compared to healthy controls, along with downregulation of multiple enzymes involved in sphingolipid metabolism. They also identified SNPs in eight genes associated with increased T1D risk. Using cis-eQTLs to assess the effect of these SNPs on gene expression, they found a significant association between *ORMDL3* and T1D (*P* < 0.05), suggesting that *ORMDL3* may play a role in genetic susceptibility to T1D (Holm et al., 2018). Yang et al. demonstrated that *ORMDL3* mRNA levels were significantly lower in peripheral blood leukocytes of children with T1D compared to healthy children. In contrast, islets from NOD mice showed a mild increase in *Ormdl3* levels during the pre-diabetic phase, potentially activating the downstream transcription factor ATF6 to promote β-cell proliferation as an early compensatory mechanism. However, as the disease progressed, *Ormdl3* expression gradually decreased (Yang et al., 2019). On the other hand, Frohnert et al. developed and compared disease risk models, showing that a weighted model incorporating ten factors, including ORMDL3, could effectively predict diabetes risk in both the general population and among first-degree relatives of T1D patients (Frohnert et al., 2018).

### 3.2 Immune system diseases

The pathogenesis of immune system diseases is complex, often involving genetic susceptibility, environmental factors, and dysregulated immune responses. In the presence of genetic predisposition, specific environmental triggers such as infections or drug exposures can induce abnormal immune reactions to self-antigens, leading to tissue damage. PBC is an autoimmune liver disease with a significant genetic component, and its development closely links to these mechanisms. Dong et al. conducted a study involving 1070 PBC patients and 1,198 controls, identifying a significant association between the rs9303277 variant in the 17q12-21 region and PBC (*P* < 0.01) (Dong et al., 2015). Xiang et al. reported that metabolically active ORMDL3+ cholangiocytes play an important immunomodulatory role in PBC pathogenesis. These cells exhibited significantly higher metabolic activity scores compared to ORMDL3- cholangiocytes. Further analysis revealed 77 differentially expressed genes between ORMDL3+ and ORMDL3- populations, which were primarily enriched in functional terms and signaling pathways related to autoimmune diseases (Xiang et al., 2021).

Beyond PBC, altered ORMDL3 expression is also linked to the risk of other immune-mediated conditions, including AS and RA. Qiu et al. identified five SNPs (rs7216389, rs12603332, rs12936231, rs9303277, and rs11557467) within a chromosomal segment at 17q21 containing *ORMDL3*, *GSDMB*, *ZPBP2*, and *IKZF3*. These SNPs showed significant association with AS (all *P* ≤ 0.01), with a particularly strong association observed in male subjects (all *P* < 0.001) (Qiu et al., 2013a). Laukens et al. also found an association between the rs2872507 locus, which was previously linked to asthma and known to affect ORMDL3 expression in lymphoblastoid cells, and AS (*P* = 0.03) (Laukens et al., 2010). Thalayasingam et al. discovered that among RA risk loci, genes stably expressed in both CD4^+^ T cells and B lymphocytes under cis-eQTL regulation included *FADS1*, *FADS2*, *BLK*, *FCRL3*, *ORMDL3*, *PPIL3*, and *GSDMB* (Thalayasingam et al., 2018). Further studies showed that the T allele of rs56199421, located within an enhancer region of ORMDL3, is associated with RA susceptibility. This allele enhances the binding affinity for the transcription factor JunD, exhibits higher transcriptional activity, and leads to increased *ORMDL3* expression in RA patients (Ye et al., 2023). Kurreeman et al. confirmed through bioinformatic analysis that the *IKZF3-ORMDL3-GSDMB* locus at 17q12 is the most significantly associated genetic region with RA (Kurreeman et al., 2012). Moreover, Dytiatkovskyi et al. observed that the C/C genotype at the *ORMDL3* rs7216389 locus reduced the risk of a specific Atopic Dermatitis phenotype, while the C/T genotype increased susceptibility (Dytiatkovskyi et al., 2021). The progressive destruction of thyroid follicles is a hallmark feature of HT, while dysregulated sphingolipid metabolism is considered a key factor in disrupting membrane lipid homeostasis and cellular stability (Dubot et al., 2025). Recent studies revealed that the *ORMDL3* rs8076131-GG/AG genotype offers a protective effect against HT by maintaining normal sphingolipid metabolism, thus preventing sphingolipid metabolic dysregulation-induced HT (Li X. et al., 2025). Collectively, these studies provide compelling evidence of *ORMDL3*’s role in immune system diseases. They enhance the understanding of disease mechanisms and suggest that targeting *ORMDL3* expression could be a promising strategy for therapeutic intervention.

### 3.3 Immune-metabolic cross-diseases

Traditionally, immunity and metabolism were regarded as relatively independent physiological systems. However, growing evidence has revealed dynamic bidirectional interactions between these systems. Sphingolipid metabolism acts as a critical hub in this network, with metabolites like Cer serving not only as energy carriers but also as key signaling molecules that regulate immune cell fate and function (Malicevic et al., 2025). The functional complexity of ORMDL3, a regulatory factor in sphingolipid metabolism, stems from its central role in this interconnected system. Obesity and asthma share a clear mechanistic link, now recognized as a distinct asthma phenotype. In obesity, adipose tissue-induced hypoxia activates HIF-1α, worsening insulin resistance and inflammation. These factors enter systemic circulation, leading to a low-grade chronic inflammatory state (Zhou et al., 2025). Inflammatory signals then propagate to the airways, stimulating airway epithelial and smooth muscle cells to release inflammatory mediators. This worsens congestion, edema, and airway hyperresponsiveness while regulating the recruitment of inflammatory cells like eosinophils and neutrophils. Ultimately, this process contributes to the heterogeneous inflammatory phenotype seen in obesity-associated asthma (Wu, 2021; Hammad and Lambrecht, 2021). Obesity also causes adipose tissue accumulation in the thoracic and abdominal regions, reducing functional residual capacity and increasing pressure on the diaphragm. This restricts lung expansion, leading to airflow limitation and airway hyperresponsiveness (Wang S. et al., 2025). Liu et al. demonstrated that in an obese asthmatic mouse model, ORMDL3 protein expression significantly increased in lung tissue. This upregulation activates the CTSD/NLRP3/GSDMD signaling axis, inducing pyroptosis and driving airway remodeling in obesity-associated asthma (Liu et al., 2023). Both diabetes and IBD involve immune-metabolic disturbances and intestinal barrier dysfunction. Diabetic model rats exhibit structural changes, such as intestinal villus atrophy and reduced goblet cells, along with a significant increase in ORMDL3 expression, which correlates positively with ATF6, suggesting its involvement in high-glucose-induced intestinal stress response. Additionally, the expression of autophagy-related genes is higher in female rats, indicating sex-based differences. These changes closely overlap with the pathological mechanisms of IBD, suggesting that ORMDL3 may serve as a common molecular link between the two diseases and a potential therapeutic target by regulating ERS and autophagy processes (Malicevic et al., 2025).

**FIGURE 3:**
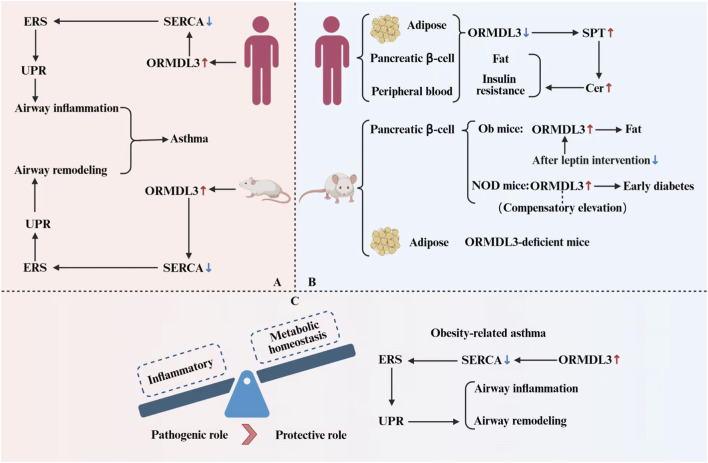
Distinct roles of ORMDL3 in asthma, obese asthma, and metabolic diseases. **(A)** In asthma, ORMDL3 inhibits *de novo* sphingolipid synthesis, thereby activating ERS and UPR, promoting the expression of downstream factors, and ultimately driving airway inflammation and remodeling. **(B)** In metabolic diseases, although ORMDL3 expression differs between humans and mice, it still plays a protective role. **(C)** In obese asthma, a classic immune-metabolic disease, elevated ORMDL3 expression shifts the balance from metabolic protection toward pro-inflammation, indicating that its disease-promoting effect predominates.

Notably, ORMDL3 exhibits contrasting expression patterns and functions in obese versus asthmatic pathological contexts. In adipose tissue, ORMDL3 helps maintain metabolic homeostasis by inhibiting Cer accumulation. In contrast, within the airway inflammatory environment, its increased expression exacerbates pyroptosis and inflammatory responses. The molecule’s metabolically protective role in obesity and its immune-disruptive function in asthma are not contradictory. Rather, they highlight ORMDL3’s ability to bidirectionally regulate immune-metabolic equilibrium across different tissue microenvironments. This tissue-specific regulatory mechanism offers new insights into the molecular connections between endocrine and inflammatory diseases and lays the groundwork for precise therapeutic strategies targeting sphingolipid metabolism. However, given the broad and significant pathophysiological impact of obesity-related asthma, the disease-promoting function of ORMDL3 appears to dominate (Figure 3).

We propose the following hypothesis: the function of ORMDL3 is not inherently paradoxical but is critically dependent on the specific microenvironment in which the cell resides. Its ultimate effect, whether protective or detrimental, is largely determined by tissue-specific signaling contexts. In adipose tissue, its expression and activity likely respond to insulin signaling and the need to maintain metabolic homeostasis, contributing to protective pathways such as inhibiting Cer accumulation. In contrast, in airway epithelium, pro-inflammatory cytokines drive its expression. The function of ORMDL3 may also change dynamically based on cell type and disease stage. In the complex pathology of coexisting obesity and asthma, metabolic and inflammatory signals intertwine, and the chronic inflammatory environment may highlight ORMDL3’s pro-inflammatory role in the disease phenotype. This hypothesis has significant theoretical implications. It goes beyond the simplistic binary classification of ORMDL3 function as purely beneficial or harmful, providing an integrative framework for understanding the molecule’s multifaceted roles across different diseases. Therefore, future therapeutic strategies targeting ORMDL3 must account for its highly context-dependent functionality. An ideal intervention should precisely modulate its harmful effects in pathological settings while preserving its protective roles in physiological conditions or other tissues. This concept lays the groundwork for developing precision treatment strategies based on sphingolipid metabolism. However, this hypothesis remains to be validated, and systematic *in vitro* and *in vivo* experiments are needed to confirm it and help transition ORMDL3-targeted therapies from theory to clinical application.

### 3.4 Circulatory system diseases

The *ORMDL3* gene, located on chromosome 17q21, was initially recognized for its strong association with childhood asthma. In recent years, multi-omics studies have increasingly highlighted its significant role in circulatory diseases, including atherosclerosis and heart failure. Zhou et al. compared the genetic correlations between asthma and nine circulatory diseases, finding a significant genetic link between asthma and heart failure. Genetic susceptibility to asthma significantly increased the risk of developing heart failure (Rg = 0.278, *P* < 0.05). The underlying mechanism may involve ORMDL3 regulating the ATF6α-ERS-Beclin1 autophagy signaling pathway, which promotes splenic B cell survival (Zhou et al., 2022). These B cells can then activate the heart-spleen axis through MHC II-dependent antigen presentation, cytokine secretion, and potential autoantibody production, which triggers the migration of immune cells from the spleen to the heart, exacerbating cardiac function (Bermea et al. 2022). Wang et al. used bioinformatic analysis to identify 15 hub genes, including *ORMDL3*, associated with ischemic stroke and myocardial infarction. These genes are involved in key biological processes such as neutrophil degranulation, neutrophil activation in immune responses, and cytokine secretion. Their study provides a new theoretical basis and potential targets for diagnosing and treating ischemic stroke and myocardial infarction (Wang E. et al., 2024). As a key member of this gene set, *ORMDL3* further emphasizes its potential regulatory role in circulatory diseases. Ma et al. confirmed that the rs7216389 T allele and the rs9303277 C allele in the *ORMDL3* gene are significantly associated with atherosclerosis risk in a Chinese Han population. In the northern population, the odds ratios were as follows: rs7216389: OR = 0.80, 95% CI (0.69–0.93); rs9303277: OR = 0.84, 95% CI (0.73–0.97). In the southern population, the values were: rs7216389: OR = 0.82, 95% CI (0.71–0.95); rs9303277: OR = 0.84, 95% CI (0.72–0.97). Patients carrying the risk alleles of rs7216389 and rs9303277 showed significantly increased *ORMDL3* mRNA and protein expression (*P* < 0.05). Further research indicated that ox-LDL can upregulate ORMDL3 expression in endothelial cells. Knocking down *ORMDL3* not only attenuated ox-LDL-induced autophagy but also suppressed the expression of the autophagy initiation protein BECN1. These findings suggest that ORMDL3 plays a crucial role in the pathogenesis of atherosclerosis by regulating autophagy in endothelial cells (Ma et al., 2015). Despite these insights, research on *ORMDL3* in the circulatory system remains limited. Most current evidence comes from population genetic association studies, with little in-depth preclinical mechanistic exploration and clinical validation. Therefore, *ORMDL3* holds significant potential to expand from an asthma susceptibility gene to a novel target for the precise prevention and treatment of circulatory diseases.

### 3.5 Digestive system diseases

Recent advances in understanding the pathogenesis of digestive diseases have increasingly highlighted ORMDL3’s key role in several gastrointestinal conditions, particularly in IBD. IBD encompasses chronic intestinal inflammatory conditions of unknown origin, primarily including UC and CD. In IBD pathogenesis, autophagy interacts closely with three core cellular pathways: intracellular bacterial infection, ERS and the UPR, and Paneth cell function. These processes collectively affect the functional homeostasis of intestinal epithelial cells (Kaser and Blumberg, 2011). A study by Skieceviciene et al. involving a Lithuanian-Latvian cohort of 444 UC patients and 1,154 healthy controls confirmed an association between the *ORMDL3* gene locus and UC. SNP-SNP interaction analysis revealed that combined effects of SNPs in the *PTPN22* (rs2476601) and *C13orf31* (rs3764147) genes increased UC risk. These findings provide new evidence for genetic susceptibility to UC and suggest potential targets for future diagnostics and therapeutics based on genetic markers (Skieceviciene et al., 2013). Research by Sharma et al. showed that ORMDL3 promotes inflammatory responses in IBD by regulating ER-mitochondria contact sites and mitochondrial dynamics. Overexpression of ORMDL3 induced mitochondrial fragmentation, enhanced ER-mitochondria interactions, and activated the NLRP3 inflammasome, exacerbating inflammation. Under inflammatory conditions, ORMDL3 also undergoes altered subcellular localization, redistributing to mitochondrial-associated membranes and mitochondria, where it interacts with the mitochondrial dynamics protein Fis-1 (Sharma et al., 2024). Malicevic et al. demonstrated that ORMDL3 accelerates IBD progression through various mechanisms, including promoting inflammation, impairing mitochondrial function, and disrupting autophagy. These insights offer new directions for developing IBD treatment strategies (Malicevic et al., 2024). Moreover, Brown et al. revealed a sex-specific role of ORMDL3 in the development of NASH. Specifically, *ORMDL3* overexpression was identified as a significant risk factor for NASH in obese males, while no similar association was observed in females. This finding sheds new light on the pathogenesis and sexual dimorphism of NASH, suggesting that gender-specific factors should be considered in future therapeutic strategies (Brown et al., 2024a). Although research on ORMDL3 in the digestive system is still in its early stages, current evidence supports its potential as a therapeutic target and provides clear directions for future studies.

### 3.6 Neurological diseases

Research into the complex mechanisms of neurological diseases has increasingly highlighted ORMDL3’s potential role. The myelin sheath, a lipid-rich structure surrounding neuronal axons, accelerates nerve signal transmission while providing electrical insulation. Clarke et al. demonstrated that during sciatic nerve myelination, ORMDL3 maintains metabolic homeostasis by inhibiting bioactive sphingolipid metabolites. A lack of finely regulated ORMDL3-mediated sphingolipid synthesis results in severe myelination defects (Clarke et al., 2019). In Alzheimer’s Disease, ORMDL3 activates the PERK/ATF4/HSPA5 signaling axis, promoting oxidative stress and inducing ferroptosis. These findings suggest that ORMDL3 could be a novel molecular target for AD-related ferroptosis and offer a potential strategy for intervention (Shao et al., 2023). In Niemann-Pick Disease Type C1, loss of the NPC1 or inhibition of its sterol-binding domain promotes *de novo* sphingolipid synthesis. Interestingly, despite elevated ORMDL expression and Cer levels, less ORMDL protein binds to the SPT-ORMDL complex. Instead, ORMDL colocalizes with the selective autophagy receptor p62 and accumulates in stalled autophagosomes due to defective autophagy in NPC1-diseased cells. Restoring autophagy flux with N-acetyl-L-leucine reduces ORMDL retention in autophagosomes and decreases *de novo* sphingolipid synthesis. This reveals a previously unknown link between sphingolipid synthesis, ORMDL function, and defective autophagy in Niemann-Pick Disease Type C1, offering new mechanistic insights and therapeutic perspectives (Brown et al., 2024b). Stefanović et al. found that reduced mRNA levels of *ORMDL3* and *GSDMB* were significantly associated with the rare homozygous TT genotype of rs12946510 in Multiple Sclerosis patients (Stefanović et al., 2024). Moreover, *ORMDL3* has been identified as a gene linked to Bipolar disorder type II and Early-onset myasthenia gravis (Can et al., 2025; Handunnetthi et al., 2021). As a pleiotropic gene, *ORMDL3* influences multiple neurological diseases through its encoded protein’s roles in regulating sphingolipid metabolism and inducing ERS. Developing targeted therapies against ORMDL3 or its downstream pathways could provide new treatment strategies for these currently incurable neurological conditions.

### 3.7 Cancer and tumours

ORMDL3 has emerged as a multifunctional regulatory factor, playing a significant role in oncology beyond its involvement in immune and inflammatory diseases. It regulates key cellular processes such as ERS, sphingolipid metabolism, EMT, and apoptosis. Its expression is linked to tumor proliferation, invasion, and metastasis. In a C57BL/6 mouse subcutaneous allograft tumor model, inhibiting *Ormdl3* increased the proportion of cytotoxic CD8^+^ T cells in the tumor microenvironment and promoted IFN-γ production, enhancing the host’s anti-tumor immune response (Zeng et al., 2025). Sun et al. found that silencing *ORMDL3* in Hepatocellular Carcinoma cells enhanced the inhibitory effects of sorafenib on cell viability and proliferation, increasing the cells’ sensitivity to the drug. *ORMDL3* knockdown elevated intracellular reactive oxygen species levels by inhibiting autophagy, which promoted sorafenib-induced apoptosis. Mechanistically, *ORMDL3* silencing inhibited the PERK-ATF4-Beclin1 signaling pathway, suppressing autophagy and improving sorafenib efficacy. In a nude mouse xenograft model, *Ormdl3* knockdown also enhanced the tumor-suppressive effects of sorafenib. This study is the first to reveal the regulatory relationship between *ORMDL3*, autophagy, and sorafenib resistance, providing important evidence for identifying synergistic therapeutic targets for sorafenib (Sun et al., 2022). Dobbins et al. confirmed that the SNP rs7216389, located in the 3′ flanking region of the *ORMDL3* gene and previously linked to childhood asthma, is also significantly associated with an increased risk of glioma (OR = 1.10, 95% CI: 1.01–1.19). This suggests a potential genetic link between asthma susceptibility and glioma risk (Dobbins et al., 2011). Yu et al. identified 194 unique pleiotropic genes in a study on acute B-cell lymphoblastic leukemia, including important genes such as *IKZF1*, *GATA3*, *IKZF3*, *GSDMB*, and ORMDL3 (Yu et al., 2024). A study on lncRNA SOX21-AS1 revealed a novel upstream regulatory pathway of *ORMDL3* in cancer. SOX21-AS1 acts as a molecular sponge for miR-520a-5p, upregulating *ORMDL3* expression and forming a critical oncogenic axis that drives the progression of TNBC. This finding not only expands our understanding of the lncRNA regulatory network but also suggests that targeting the SOX21-AS1/miR-520a-5p/*ORMDL3* axis could represent a new therapeutic strategy for TNBC (Liu et al., 2020). In summary, ORMDL3 plays a crucial regulatory role in multiple cancer types. Its potential as a therapeutic target and prognostic biomarker is increasingly recognized.

### 3.8 Other diseases

ORMDL3 demonstrates diverse biological functions across various pathological contexts, offering new perspectives on disease pathogenesis and potential therapeutic strategies. Using Transcriptome-wide Association Studies, Wang et al. identified shared genes associated with both chronic rhinosinusitis and autoimmune diseases. Notably, the SNP rs11557467 in the *ORMDL3* gene showed strong associations with Atopic Dermatitis, Allergic Rhinitis, and Chronic Rhinosinusitis, suggesting *ORMDL3* as a common genetic factor linking multiple immune-related diseases (Wang M. et al., 2024b). ORMDL3 influences rhinovirus infection and inflammatory responses through several mechanisms. It upregulates ICAM-1, a major rhinovirus receptor in epithelial cells, and promotes ceramide-enriched membrane structures that enhance viral entry and replication. Additionally, ORMDL3 modulates ERS, ceramide, and S1P levels, leading to pro-inflammatory cytokine release, which increases rhinovirus infectivity and inflammation (Ito and Zhang, 2020). Chang et al. showed that the expression of *Ormdl3* and *Zpbp2* is regulated by circadian rhythms and is tissue-specific. In the lung and ileum, deletion of *Zpbp2* affects Nr1d1 expression in a time-dependent manner, while in the liver, it increases *Ormdl3* expression (Chang et al., 2019). Respiratory syncytial virus is a major cause of bronchiolitis in infants, which can lead to recurrent wheezing and asthma. After infection, IRF-3 binds directly to the *ORMDL3* promoter, playing a key role in its expression, which may contribute to inflammation in bronchiolitis and wheezing (Wang et al., 2017). In a study involving 247 infants with bronchiolitis and 190 controls, Liu et al. found that the TT homozygous genotype and T allele of the rs7216389 locus in *ORMDL3* were associated with an increased risk of bronchiolitis (*P* < 0.05). This suggests that the rs7216389 polymorphism could be a biomarker for identifying infants susceptible to virus-induced wheezing and asthma (Liu et al., 2015). In summary, ORMDL3 is a key regulatory factor in a variety of diseases and physiological processes, including circadian rhythm regulation. From genetic polymorphisms to expression regulation, and from molecular mechanisms to clinical phenotypes, ORMDL3 plays a central role in complex disease networks. These findings not only enhance our understanding of disease mechanisms but also provide a foundation for developing targeted intervention strategies. Further studies and clinical validation will help clarify its full biological significance and support its translational applications.

## 4 Summary and outlook

ORMDL3 is a pleiotropic regulatory factor, initially identified as an asthma susceptibility gene, but its role extends to a variety of diseases. Recent evidence highlights its critical involvement in the development and progression of asthma, metabolic diseases, circulatory diseases, and cancer. This review summarizes the expression patterns, molecular mechanisms, and clinical significance of ORMDL3 across different diseases and outlines future research directions. ORMDL3 plays a central role in disease pathogenesis by regulating key processes like sphingolipid metabolism, ERS, UPR, autophagy, inflammatory responses, and cell death. In asthma, ORMDL3 is involved in airway inflammation and remodeling. In metabolic diseases, it regulates insulin signaling and lipid metabolism, and in circulatory diseases, it may influence atherosclerosis and heart failure. Notably, in oncology, ORMDL3 modulates tumor cell proliferation, invasion, metastasis, and drug sensitivity. It also has potential immunomodulatory effects within the tumor microenvironment, making it a promising therapeutic target in cancer.

**FIGURE 4:**
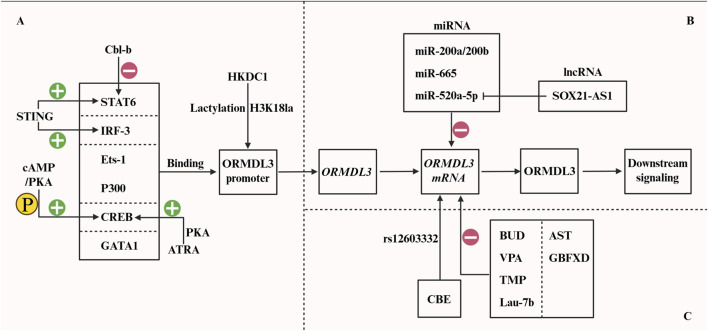
Multi-pronged interventions targeting ORMDL3. **(A)** Transcription factors STAT6 (Qiu et al., 2013b), IRF-3 (Wang et al., 2017), Ets-1 (Jin et al., 2012), p300 (Cheng et al., 2019), CREB (Zhuang et al., 2013a), and GATA1 (Chen et al., 2025) can directly bind to the promoter region of ORMDL3. Its upstream signals also exert a certain regulatory effect. STING positively regulates STAT6 and IRF-3 (Cao et al., 2018), while cbl-b exerts a negative regulatory effect on STAT6 (Yang et al., 2015). cAMP activates PKA, which in turn phosphorylates CREB, promoting its binding to the ORMDL3 promoter (Zhuang et al., 2013a). Furthermore, HKDC1 promotes the lactylation of H3K18 at the ORMDL3 promoter (Zhang et al., 2026). **(B)** At the mRNA level, miR-200a/200b (Duan et al., 2020), miR-665 (Li et al., 2017), and miR-520a-5p (Liu et al., 2020) can negatively regulate the expression of *ORMDL3*. **(C)** A variety of commonly used methods can downregulate *ORMDL3* mRNA expression, including BUD (Yu et al., 2017), VPA (Zhang et al., 2017), TMP (Ding et al., 2021), Lau-7b (Youssef et al., 2020), as well as the compound Chinese medicine GBFXD (Lu et al., 2016) and AST (Xiang et al., 2019). ATRA promotes the binding of CREB to the CRE element in the ORMDL3 promoter region and initiates transcription by inducing PKA-dependent CREB phosphorylation (Zhuang et al., 2013b). Furthermore, the use of CBE for single-base editing of rs12603332 can modulate ORMDL3 expression (Weng et al., 2022).

Therapeutically, inhibiting P300 with agents like C646 specifically disrupts its acetylation of the *Ormdl3* promoter, downregulating *Ormdl3* expression and alleviating asthma symptoms in models (Cheng et al., 2019). The fenretinide formulation Lau-7b protects by suppressing allergen-induced *Ormdl3* overexpression, correcting the imbalance between very long-chain and long-chain ceramides (VLCCs/LCCs), and reducing core asthma symptoms such as airway hyperresponsiveness and inflammation (Youssef et al., 2020). Further evidence suggests that upregulating miR-200a and miR-200b could suppress asthmatic inflammation by preventing the activation of the ERK/MMP-9 signaling pathway, which triggers pro-inflammatory cytokine production (Duan et al., 2020). Notably, AST has been shown to downregulate *Ormdl3* expression in asthmatic models. This reduces sphingolipid metabolic disruptions and inhibits NF-κB signaling, which decreases inflammation and collagen deposition (Xiang et al., 2019). See the figure below for details (Figure 4). These diverse strategies, ranging from gene regulation to traditional medicine, target ORMDL3, improving airway inflammation and remodeling. While promising, developing drugs targeting ORMDL3 faces challenges. Systemic inhibition may interfere with essential cellular functions, leading to off-target effects or toxicity. Furthermore, creating selective small-molecule inhibitors or gene therapies to target specific cell types is critical for safety and efficacy but remains technologically challenging. The complexity of ORMDL3-related pathways and compensatory mechanisms also limits single-target therapies and may promote resistance. Despite these hurdles, research on ORMDL3-targeted drugs is advancing, offering a pathway for overcoming obstacles and developing next-generation asthma therapies.

Although significant progress has been made in ORMDL3 research, its mechanisms and clinical applications remain challenging. ORMDL3 may have distinct or even opposite functions depending on the tissue and disease context, indicating a complex regulatory network that is not yet fully understood. Additionally, most evidence comes from GWAS or animal models, with a lack of direct human clinical data, limiting its translation into clinical practice. Moreover, ORMDL3 collaborates with genes like *GSDMB* and *IKZF3*, and their functional relationships need further study. To address these challenges, future research should focus on several key areas: understanding the tissue- and disease-specific regulatory mechanisms of ORMDL3, particularly its molecular networks and signaling pathways; developing selective ORMDL3 inhibitors or therapeutic strategies targeting its downstream effectors for new drug development; conducting large-scale population-based cohort studies and genetic analyses to assess the potential of ORMDL3 as a biomarker for early diagnosis, risk prediction, or treatment evaluation; and exploring ORMDL3’s applications in disease prevention, combination therapies, and personalized medicine.

**FIGURE 5:**
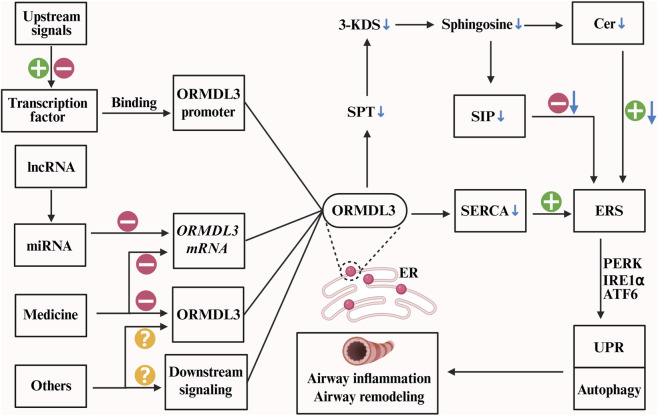
Integrated model featuring ORMDL3 as a central regulatory node.

In conclusion, ORMDL3 is not only an important disease-associated molecule but also a key regulatory node with multifaceted biological functions (Figure 5). In-depth research on ORMDL3 is expected to provide new theoretical foundations and therapeutic strategies for the prevention and treatment of various diseases, including asthma, metabolic diseases, circulatory diseases, and cancer.

## Glossary

Acyl-CoA: Fatty acyl-CoA
AP-1: Activator Protein-1
AS: Ankylosing Spondylitis
AST: Acupoint Sticking Therapy
ATF: Activating Transcription Factor
ATG16L1: Autophagy-related 16-like 1
ATG3/7: Autophagy-related protein 3/7
ATRA: All-Trans Retinoic Acid
BAT: Brown adipose tissue
BUD: Budesonide
cAMP: Cyclic Adenosine Monophosphate
CBE: Cytosine Base Editor
CD: Crohn’s Disease
Cdase: Ceramidase
Cer: Ceramide
CerS: Ceramide Synthase
CHOP: C/EBP Homologous Protein
CREB: cAMP Response Element-Binding Protein
DHS: Dihydrosphingosine
DHSDH: Dihydrosphingosine Dehydrogenase
ECM: extracellular matrix
EMT: epithelial-mesenchymal transition
eIF2α: Eukaryotic Initiation Factor 2 Alpha
ER: Endoplasmic Reticulum
ERAD: ER-Associated Degradation Protein
ERK: Extracellular Signal-Regulated Kinase
ERS: Endoplasmic Reticulum Stress
Ets-1: Ets-1 Transcription Factor
FcεRI: Fc epsilon receptor I
GATA: GATA Binding Protein
GBFXD: Guben Fangxiao Decoction
GSL: Glycosphingolipid
GWAS: Genome-Wide Association Study
HIF-1α: Hypoxia-Inducible Factor-1 alpha
HKDC1: Hexokinase Domain Containing 1
HSPA5: Heat Shock Protein Family A Member 5
HT: Hashimoto’s thyroiditis
IBD: Inflammatory Bowel Disease
ICAM-1: Intercellular Adhesion Molecule-1
IFN-γ: Interferon-gamma
IL: Interleukin
IP3R: Inositol 1,4,5-trisphosphate Receptor
IRE1α: Inositol-Requiring Enzyme 1 Alpha
IRF-3: Interferon Regulatory Factor 3
JNK: c-Jun N-terminal kinase
KDSR: 3-Ketodihydrosphingosine Reductase
LC3: Microtubule-associated protein 1 light chain 3
lncRNA: Long Non-Coding RNA
MCP-1: Monocyte Chemoattractant Protein-1
MHC II: Major Histocompatibility Complex Class II
miR: MicroRNA
MMP-9: Matrix Metalloproteinase-9
NASH: Non-alcoholic Steatohepatitis
NF-κB: Nuclear Factor-kappa B
NPC1: Niemann-Pick C1
*ORMDL3*: *Orosomucoid-like 3*
ox-LDL: Oxidized Low-Density Lipoprotein
Pal-CoA: Palmitoyl-CoA
PBC: Primary Biliary Cholangitis
P-ERK: Phosphorylated ERK
PERK: PKR-like ER Kinase
PKA: Protein Kinase A
P300: Histone Acetyltransferase p300
p38 MAPK: p38 mitogen-activated protein kinase
RA: Rheumatoid Arthritis
RIDD: Regulated IRE1-Dependent Decay
RyR: Ryanodine Receptor
SERCA: Sarco/ER Calcium ATPase
SM: Sphingomyelin
SMS: Sphingomyelin Synthase
SNPs: Single nucleotide polymorphisms
SOX21-AS1: SOX21 Antisense RNA 1
SPT: Serine Palmitoyltransferase
STAT: Signal Transducer and Activator of Transcription
STING: Stimulator of interferon gene
S1P: Sphingosine-1-Phosphate
TMP: Tetramethylpyrazine
TNBC: Triple-negative Breast Cancer
TNF-α: Tumor Necrosis Factor-alpha
T1D: Type 1 diabetes
UC: Ulcerative Colitis
UCP1: Uncoupling Protein 1
UPR: Unfolded Protein Response
VPA: Valproic acid
WAT: White adipose tissue
XBP1: X-box Binding Protein 1
3-KDS: 3-Ketodihydrosphingosine
